# Same, same, but different? A longitudinal, mixed-methods study of stability in values and preferences for future end-of-life care among community-dwelling, older adults

**DOI:** 10.1186/s12904-021-00839-7

**Published:** 2021-09-22

**Authors:** Malin Eneslätt, Gert Helgesson, Carol Tishelman

**Affiliations:** 1 Department of Learning, Informatics, Management and Ethics, Karolinska Institutet, 171 77 Stockholm, Sweden; 2 Department of Health, Education and Technology, Luleå University of Technology, Luleå, Sweden; 3 Stockholm Health Care Services (SLSO), Region Stockholm, Stockholm, Sweden; 4 University of Southampton, School of Health Sciences, Southampton, UK

**Keywords:** Advance care planning, End-of-life care, End-of-life conversations, Values and preferences, Mixed-methods, DöBra, Go Wish, Go-wish

## Abstract

**Background:**

End-of-life preferences may change over time, e.g. due to illness progression or life events. Research on stability of end-of-life preferences has largely focused on life-sustaining treatments in seriously ill patients or medical decision-making based on hypothetical illness scenarios and possible treatment options. Few studies focus on community-dwellers in natural settings. The aim of this study was thus to explore if and how community-dwelling, older adults’ prioritizations and reasoning about values and preferences for future end-of-life care change over time.

**Methods:**

Using a mixed-methods design, we explored stability of end-of-life preferences in older community-dwelling adults without imminent end-of-life care needs. At two timepoints (T1 and T2), 5.5–12 months apart, 52 individuals discussed what would be important to them at the end-of-life, through open conversations and while using DöBra cards, a Swedish version of GoWish cards. Participants ranked their most important card statements from 1 to 10. Stability in card rankings, i.e. a card recurring in the top-10 ranking at T2 regardless of position, was explored using descriptive statistics and non-parametric analyses. Participants’ reasoning about card choices were explored with longitudinal qualitative analysis.

**Results:**

Stability between T1 and T2 in the top-10 priorities ranged from 20 to 80%, median 60%. Stability in cards rankings could not be explained by changes in participants’ health status, extent of card use (no/little/frequent use) between interviews, or days between T1 and T2, nor was it related to demographic variables. Qualitative analysis showed that consistent reasoning was not always paired with consistency in card choices and changed card choices were not always related to changes in reasoning.

**Conclusions:**

Longitudinal exploration combining DöBra card rankings with underlying reasoning about end-of-life preferences over time furthers knowledge on the dynamics between values and preferences in end-of-life decision-making. Individuals’ end-of-life preferences in form of card choices were relatively stable over time albeit with large variation between different individuals. However, the values and underlying reasoning that participants used to motivate their choices appeared more stable than ranking of card choices. We thus conclude that concurrent conversation-based exploration is a more comprehensive indicator of end-of-life values and preferences over time than ranking of cards alone.

**Supplementary Information:**

The online version contains supplementary material available at 10.1186/s12904-021-00839-7.

## Background

The European Association for Palliative Care has broadly defined advance care planning (ACP) as a process of reflecting on and making decisions for future end-of-life care [1]. It has been reported to positively impact the quality of end-of-life care, increase the proportion of goal-concordant care at the end-of-life, and lower hospital readmission rates [2–4]. However, there is a need to better understand how stable end-of-life preferences are over time, given that they may change, e.g. due to illness progression or life events [5, 6]. Previous research on stability of preferences for future end-of-life care has largely focused on preferences for life-sustaining treatments in seriously ill patients [7–10].

A comprehensive review [11] found that individuals’ end-of-life preferences were generally stable over time as well as after changes in health status, with this tendency more pronounced among seriously ill patients and inpatients than community-dwelling adults. However, another important finding was large variability in stability of end-of-life preferences among different studies. Although most studies evaluating direction of changes in end-of-life preferences indicated wanting less aggressive medical treatment over time and as an illness progressed, some studies reported contradictory findings [11]. A longitudinal study of preferences for life-prolonging vs. comfort care among patients with advanced cancer also showed inconsistent and unpredictable changes in preferences, suggesting high individual variation [12]. Longitudinal studies of community-dwellers’ end-of-life care preferences are scarce, focusing primarily on stability of preferences for discrete medical treatments at the end-of-life [13, 14] or euthanasia [15].

In the *Advance care planning in Sweden (SweACP)* project, we have examined end-of-life values and preferences broadly. SweACP, part of the DöBra^1^ research program [16], is a nation-wide research project planned and conducted in collaboration between a transdisciplinary team of researchers and representatives of national, community-based patient and interest organizations: the Association of Relatives to Cancer Patients, the Dementia Association, the Lung Cancer Interest Organization, Network against Cancer, the Swedish Association for Senior Citizens (SPF), and the Swedish National Pensioners’ Organization (PRO). We engaged community-dwelling older adults who were not known to be at the end-of-life in conversations about what they thought would be important to them in their future end-of-life care, stimulated by a Swedish adaptation of GoWish cards [17]. In line with the card statements, we made efforts to address the overall life situation of a dying person instead of focusing solely on medical treatments. This approach was in part a product of possibilities that the ACP-naïve Swedish context presents, where documentation of end-of-life preferences in the form of advance directives is briefly mentioned in the Swedish national guidelines for palliative care [18], but ACP is rare in Swedish health care settings. ACP is furthermore virtually unheard of among the general public, and documentation of end-of-life care wishes, or appointment of proxy decision-makers is not legally binding. It was also in part a concerted choice, based on previous international research, to develop an early [19], community-based [20], conversational ACP approach to stimulate processes of reflection and continued conversation [21] rather than medical decision-making and documentation of treatment preferences.

As previous studies about stability of end-of-life preferences primarily engaged participants in medical decision-making based on pre-formulated illness scenarios and possible treatment options [13, 14], there is need for further research in natural settings [11]. The GoWish/DöBra cards support clarifying real-world values as participants reflect about their own values and preferences for future care rather than considering set hypothetical scenarios. Two studies have examined stability of preferences using the GoWish cards; however, one was limited to comparing preferences in people with and without dementia [22] and the other had a narrow measurement window, studying preferences expressed only 4–24 h apart [23].

We therefore attempt to fill several important gaps in the literature as this study involves community-dwelling older adults, an understudied population, and longitudinally explores broad end-of-life values and preferences using a translated and adapted Swedish version of GoWish cards. Furthermore, we study not only stability of ranked card preferences but also reasoning underlying these choices. This could further the understanding of how to interpret previously stated end-of-life preferences, e.g. when an individual has lost capacity to express their wishes. Providing insights into motivations behind expressed preferences can be helpful for both health care staff and families in grappling with future care decisions, even ones which were never specifically addressed when the individual had capacity. The aim of this study is thus to explore if and how community-dwelling, older adults’ prioritizations and reasoning about values and preferences for future end-of-life care change over time.

## Methods

Using a longitudinal, mixed-methods design we compared future end-of-life prioritizations and reasoning about these at two timepoints (T1 and T2) using descriptive statistics and non-parametric statistical analyses to explore stability in card rankings, and longitudinal qualitative analysis to explore changes and similarities in reasoning over time. Definitions and operationalizations of the concept of stability are furthered described under ‘Data Analysis’.

### Data collection

**Fig. 1:**
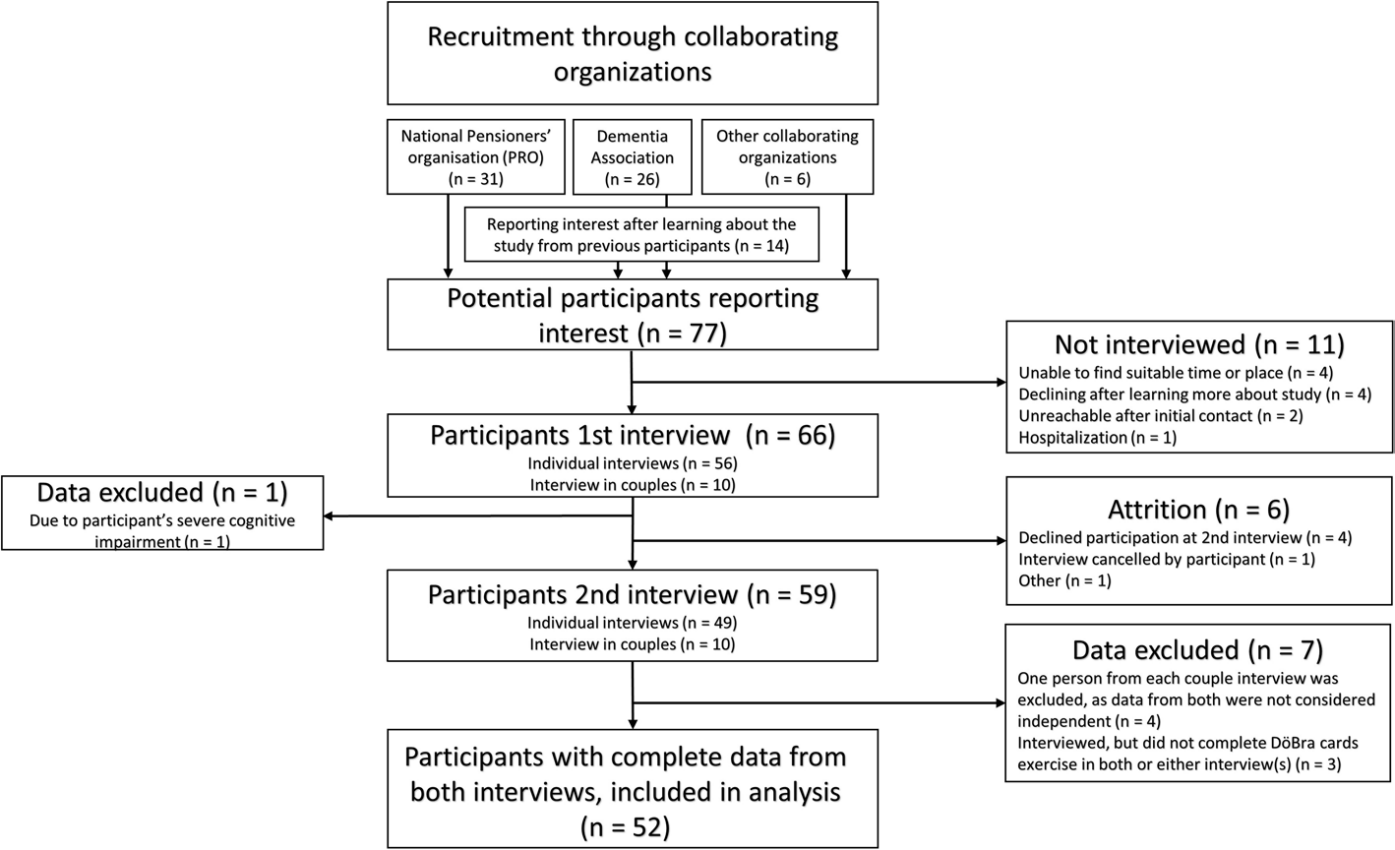
Flowchart of recruitment and participation in interviews

The SweACP project focused on engaging older, community-dwelling adults without imminent end-of-life care needs in conversations about future end-of-life care. After receiving ethical approval (Stockholm, #2015/106–31/5) study information was disseminated through the collaborating community organizations and a recruitment strategy based on active volunteering undertaken, with those interested in participating contacting the researchers. Written informed consent was provided. The only eligibility criteria were cognitive function that allowed understanding of informed consent and completion of the interview protocol, as well as being able to communicate in Swedish. Given the lack of formalized ACP initiatives in Sweden mentioned above, previous experience of end-of-life/ACP discussions was not a prerequisite. Purposeful and snowball sampling was used; a flowchart of recruitment and participation is shown in Fig. 1.

Between May 2015 and January 2018, two audio-recorded interviews were conducted with each participant 5.5 to 12 months apart. This timeframe was chosen to complement previous research [22, 23], and to allow for significant life events which may alter end-of-life values and preferences to occur, as well as reduce likelihood of participants’ rankings being based on memory from the 1st interview. Interviews were held by one of two female research assistants, previously unknown to participants, with experience in conducting research interviews and MSc in Nursing (ME) and Forensic Psychology (TJ). While conversational in nature, interviews followed similar protocols at T1 and T2. Issues of who and what participants thought would be important to them at end-of-life were initially explored through open-ended questions and subsequent prompts when needed. This was followed by a semi-structured interview section in which an EcoMap [24] was used to reflect on who would be important at the end-of-life (reported on elsewhere [25]). The GoWish cards had previously been found to be an intuitive, feasible tool to support end-of-life discussions in various settings in easing initiation of conversations by offering users something concrete to do with pre-formulated statements to consider that can be complemented by individualized ‘wild cards’ [17, 26–29]. An adapted Swedish version, the DöBra card deck, was therefore used to stimulate conversations about what would be important at the end-of-life.

With permission from and in collaboration with the developers (www.codaalliance.org), the U.S. GoWish cards were translated and adapted to Swedish conditions by the SweACP project group, a process described elsewhere [25]. The DöBra cards contain 37 statements about that which may be important at end-of-life, and ‘wild cards’ for users to freely formulate other matters of importance (see Supplementary File 1). Participants sorted DöBra cards into three piles from most to least important and then ranked their ten most important card items from 1 to 10. This ranking was photo-documented by the interviewer. Participants were encouraged to reflect on their choices throughout the exercise, thus generating data about their reasoning process while ranking card statements. Interviews were professionally transcribed verbatim and reviewed by the interviewer.

### Data analysis

A mixed-methods approach was applied to data analysis to gain a comprehensive understanding of individuals’ card rankings as well as their reasoning about their end-of-life values and preferences. We adhere to the GRAMM guidelines [30] for reporting on mixed-methods studies.

We applied a definition of stability in card rankings as a DöBra card item remaining in the individual’s top-10 ranking at both timepoints, regardless of specific rank. This has been used by comparable studies [22, 23]; and also matched our experiences from the card sorting exercise in the interviews. Wild card formulations were examined individually and judged stable when similar formulations recurred and as changed when formulations substantially differed between timepoints. The stability of each individual’s ranking was calculated as a percentage of number of recurring card items divided by the maximum number of cards ranked at either timepoint. For example, four identical card items in the top-10 rankings with 10 cards prioritized in both interviews equaled 40% stability. When an individual had not ranked 10 cards in either interview, the number of recurring card items was divided with the maximum number of cards ranked in either interview. Due to the relatively small sample size, we used non-parametric analyses – Mann Whitney U-test, Kruskal-Wallis H test and Spearman’s correlation test – to explore associations between the 52 participants’ characteristics and stability in card rankings.

Inspired by Saldaña [31], we employed longitudinal qualitative analysis to a sub-sample to explore change in data through time. Data from four of the participants with the most (80%) and the four with the least (20–33%) stable card rankings were first analyzed. We initially explored similarities in reasoning within each of these groups and whether the degree of stability in reasoning differed between groups. Since we saw little difference between groups, we continued analysis by “re-casing” in line with Sandelowski [32]. Re-casing shifted the analytic focus from comparing reasoning of individuals in groups with most/least stable rankings to comparing reasoning about specific consistent/inconsistent card choices. In addition to re-visiting data of the eight participants with high/low stability, data from four additional participants with varied stability (40–70%), varying demographical features and extensive data on reasoning processes were added to analysis at this stage.

Analysis was performed with adapted ‘analytic flip charts’ [31] in a text document, collating data for each individual, including time elapse between interviews, demographic characteristics, field notes, and top-10 DöBra card rankings, as well as interview excerpts with reasoning about the same card statement from both interviews. Using framing questions suggested by Saldaña [31], i.e. “How has ranking and reasoning changed between T1 and T2?” and “What contextual conditions seem to influence changes in reasoning?”, we compared individual participants’ reasoning about identical and changed card choices at T1 and T2. As analysis of data from these 12 participants, a sample size commonly considered adequate for qualitative analysis [33], showed clear patterns in reasoning about card choices, we did not include additional data in our in-depth qualitative analysis. However, we thereafter reviewed the full data set to ensure that the remaining data did not change or contradict the conclusions drawn based on the sub-sample. First author ME was responsible for carrying out the analysis but engaged in frequent discussions with the other authors during the process to enhance credibility.

## Results

### Sample characteristics

While 65 participants were initially interviewed in the SweACP project, 52 of them completed both interviews with full records and were included in analysis for this study, as shown in Fig. 1. Demographic information of original participants not included in analysis are shown in Supplementary file 2. Participants will in tables and text below be referred to as P for ‘participant’, followed by their interview code. Sample characteristics are shown in Table 1. Time between interviews ranged from approximately 5.5–12 months (median ~ 10 months). As shown in Table 1, participants were asked for an assessment of their health status but otherwise not specifically queried about the presence of illnesses.

**Table 1:** Sample characteristics

**Characteristics**	**Full sample (*N* = 52)** **N (%)**	**Sub-sample for qualitative analysis (*N* = 12)** **N (%)**
*Age*, median (range)	74 (43–95) yrs	75,5 (65–88) yrs
*Gender*		
Female	38 (73,1)	9 (75,0)
Male	14 (26,9)	3 (25,0)
*Living situation*		
Spouse	23 (44,2)	2 (16,7)
Alone	28 (53,8)	10 (83,3)
With children	1 (1,9)	0 (0,0)
*Education*		
University	27 (51,9)	6 (50,0)
High school	9 (17,3)	1 (8,3)
Elementary school	12 (23,1)	4 (33,3)
Other	4 (7,7)	1 (8,3)
*Employment status*		
Retired	44 (84,6)	12 (100)
Employed, part-time	4 (7,7)	0 (0,0)
Retired, working part-time	2 (3,8)	0 (0,0)
Student, full-time	1 (1,9)	0 (0,0)
Employed, full-time	1 (1,9)	0 (0,0)
*Self-assessed health status*^*a*^		
Good	37 (74,0)	7 (58,3)
Neither good nor poor	10 (20,0)	4 (33,3)
Poor	3 (6,0)	1 (8,3)

^a^Missing data for two participants in full sample

### Stability of DöBra card rankings

In this sample, median stability in card rankings between timepoints was 60%, ranging from 20 to 80%, as seen in Table 2. Only four participants had < 40% stability and 27 participants showed 60–70% stability between timepoints.

**Table 2:** Frequency table of card ranking stability (*n* = 52)

*Stability*	*Frequency*	*Valid percent*	*Cumulative percent*
20%	1	1.9	1.9
29%	1	1.9	3.8
30%	1	1.9	5.8
33%	1	1.9	7.7
40%	5	11.5	19.2
50%	8	13.5	32.7
60%	17	32.7	65.4
70%	10	19.2	84.6
80%	8	15.4	100.0
Total	52	100.0	

Sex, age, level of education, and time elapsed between interviews were not associated with stability of card rankings. We found no relationship between changes in self-reported health status between timepoints and stability of card rankings. Participants were asked in the second interview if they had used the DöBra cards since the first interview, with varying responses. No use (indicated by 42% of participants), little use (1–3 times, indicated by 23%) or more frequent use of the cards (> 3 times, indicated by 29%) between interviews was not associated with stability in rankings.

We explored if particular card statements were ranked in the top-10 in both interviews to a higher extent than others. As shown in the Supplementary File 1, the card statements that were most likely to be ranked in the top-10 in both interviews, i.e. ‘To be free of pain’, ‘Not being short of breath’ and ‘To have those I am close to around me’, were also the card statements most frequently prioritized in general. The card statement ‘Not dying alone’ was less frequently ranked in the top-10 overall, but when prioritized by an individual, it often appeared at both their timepoints. ‘To be cared for by staff I feel comfortable with’ is an example of a statement often prioritized (*n* = 34), but relatively seldom ranked in the top-10 by the same participant at both timepoints.

**Fig. 2:**
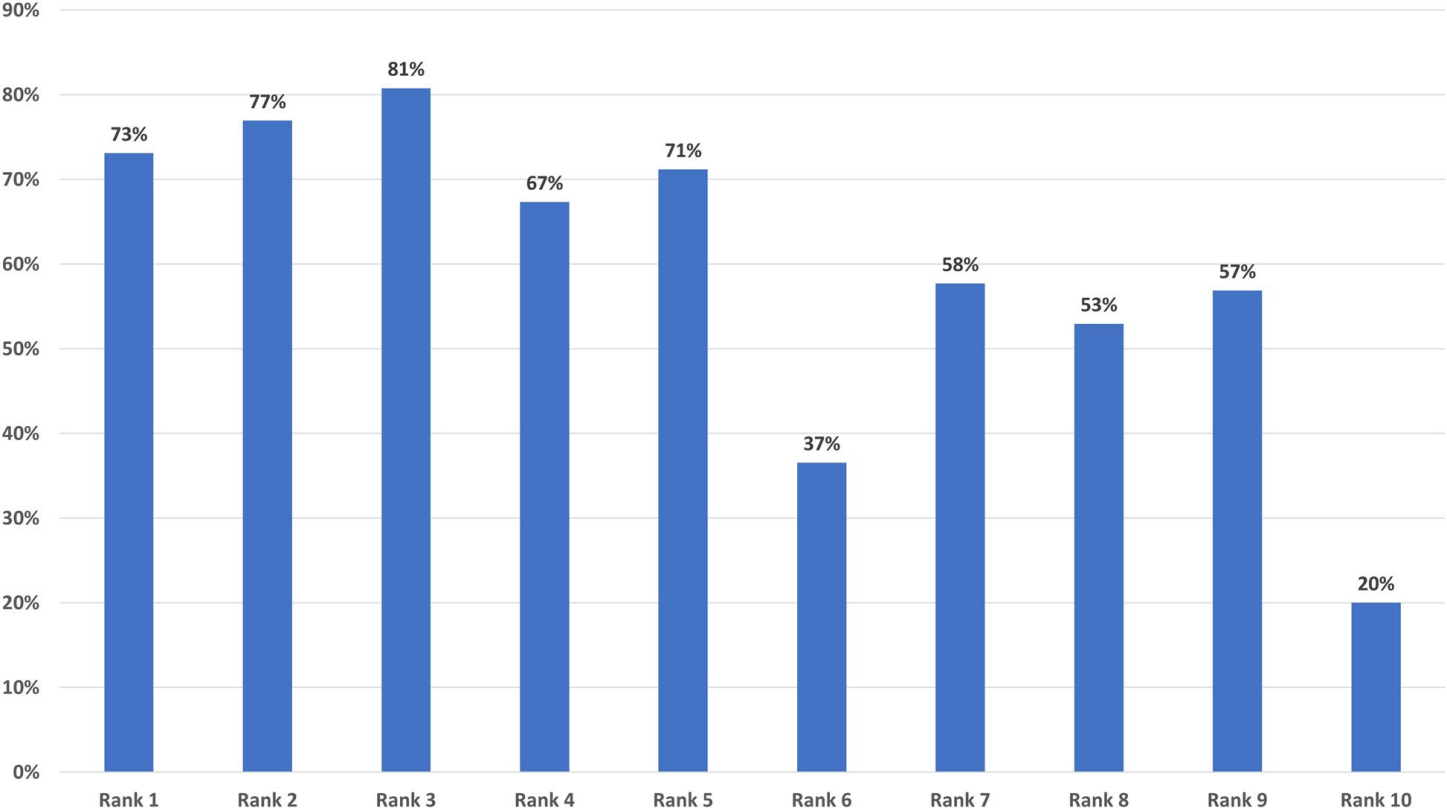
Percentages of card statements ranked 1–10 at T1 that reoccurred in the top-10 at T2, regardless of ranking order at T2 (*n* = 52)

We then examined (Fig. 2) whether a card item ranked highly among the top-10 cards at T1 would be more likely to remain in the top-10 at T2, compared to card statements that were lower ranked among the top-10 cards at T1. On a group level, cards with higher rankings at T1 were significantly more likely to recur among the top-10 priorities at T2, regardless of ranking (Spearman’s correlation coefficient − 0.86, *p* = 0.002).

### Stability of wild card formulations

Twenty-two wild cards were prioritized in the top-10 rankings by 19 individuals at T1 and 23 wild cards by 20 participants at T2, as shown in Table 3. Six individuals formulated a wild card only at T1, and seven other participants formulated a wild card at T2 only. Wild card formulations could vary over time. Nine wild cards formulated at T1 recurred exactly or with a close formulation at T2. Four participants formulated substantially different wild cards at the two timepoints (Table 3).

**Table 3:** Overview of wild cards in both interviews

**Rank (1–10). Wild card formulation.**		
**Participant #**	**First interview (T1)**	**Second interview (T2)**
P2	(10). To get help in ending my life when I want to	(1). That there is help for euthansia
P5	(10). To be able to end my life when I want to	[No wild card]
P7	(10). To know that someone will take care of my close ones	(1). That one can get help ending it all, in a dignified manner
P10	(1). To get to decide myself when to die… I’m kind of in favor of euthanasia	(1). To be entitled to euthanasia
P12	[No wild card]	(10). To be able to have passive euthanasia, to not be connected to life-sustaining machines, to stop treatment
P13	[No wild card]	(10). To get an injection and die
P14	(2). To have flowers around me	[No wild card]
P18	(1). To have room for my spirituality	[No wild card]
P19	(1). To have my teddy bear with me	(1). To be entitled to euthanasia
P20	(4). To be entitled to euthanasia	(6). To be able to communicate
P21	(2). To have the right to end my life myself	(3). The right to choose a place (5). To have the right to end my life
P25	(10). To be able to stop eating when I know the end is near	(10). To not get nutritional drinks but to let my body die naturally
P27b	(6). To be a partner for discussion in health care all the way to the end	[No wild card]
P28	(5). Not being connected to machines **when you don’t have a life with dignity**	(10). Not being connected to **life-sustaining** machines
P30	[No wild card]	(8). To not be force-fed
P33	(1). To be able to decide myself when to end my life	(5). Euthanasia
P43	[No wild card]	(4). To not have to lay there thirsty
P45	(2). To not have any life-sustaining measures	(1). Nothing left to lose [spoken in English] – I don’t want to have any of my things left
P48	[No wild card]	(4). That there are people around me, close by (7). A beautiful environment
P49	[No wild card]	(8). To be able to decide a place myself
P51	(4). To be able to eat what I like (7). To have someone take responsibility for my finances	(1). To have someone take responsibility for the finances (6). Food and drink
P53	(2). To **have** peace with God (9). To get the strength to physically and mentally support my wife	[No wild card]
P57	(1). To have the right to end my life	(1). To get euthanasia to avoid breathlessness, pain, worry and anxiety
P58	(1). To be able to give love to those I meet, until the end (2). To convey courage and confidence	(1). To be able to spread peace, love and contentment to others up to the very end
P59	(1). To have the opportunity of assisted living	[No wild card]
P61	[No wild card]	(6). To wear my own clothes

Phrases in bold indicate wild card formulations which adjusted existing DöBra card formulations. Four participants formulated five additional wild cards which were not included in their top-10 ranking and therefore not presented here

### Stability of reasoning about ranked end-of-life preferences: same, same, but different?

Characteristics of the 12 participants whose data provide basis for qualitative analysis are presented in Table 1. As elaborated on below, we found that similarities and differences in reasoning were not necessarily related to similarities and differences in ranked card choices; Table 4 shows an overview of this, while further exploration of participants’ individual reasoning is exemplified with quotes in Table 5.

**Table 4:** Overview of types of reasoning about identical/changed card choices

	**Similar reasoning**	**Changed reasoning**
**Identical card choice**	- Profound experiences - Strong opinions - Habits	- Added nuances - Modified argumentation
**Changed card choice**	- Similar reasoning that did not explain the change in card choice - Change in relative importance of card item	- Change in definition of card item - Issue had been resolved between timepoints

**Table 5:** Examples of reasoning about identical/changed card choices

**Partic-ipant #**	**Card statement**	**(Rank). Quotes illustrating reasoning at T1**	**(Rank). Quotes illustrating reasoning at T2**
***Reasoning about recurring card choices***			
P9	Not being short of breath	(4). *My uncle, he wasn’t a bad person but he believed that he… he had rock hard military discipline and when I didn’t obey, then I was locked in a warm and damp closet they used for drying laundry. And it was behind a stone wall, so it was always hot and it was pitch-dark in there. And after that I’ve always been terribly afraid of not getting air*.	(1). *I’ve always been afraid of suffocating. It started when I was a child, I was with my aunt and uncle and was locked in a warm and dark closet when he thought I was…They didn’t have children and he had principles.*
P18	To have a human touch	(2). *This with touch is very interesting. I could have a lecture for you now (laughs). But really, if you’ve read books by people who can’t take care of themselves, how you are touched… That is, physically touched when someone touches you. Warm hands, cold hands, heavyhanded and gently. You understand, so… So touch is very important.*	(6). *And to have a human touch, that’s very important. I’m trained in tactile massage* [a form of massage developed in Sweden, consisting of light touch and pressure]*, so I know what touch does with us.*
P53	To pray	(1). *Yes, since I’m religious and have prayed every day, well maybe not absolutely every day, but really mostly every day, and found it to be a…an absolute support in life.*	(2). *Yes, but just this, my faith in God and just having the possibility of having that contact and at the same time also prayer, it is… Evening prayers have become much more important as time has passed.*
P7	To be cared for by staff I feel comfortable with	(7). *And if I’m going to be cared for by staff, I want to get along with them and that they see me as the person I am.*	(8). *I really believe that we staff, and staff and those around create more problems than benefit. This with humanity, it’s important to be able to see a person and like, who am I there for. Because staff and friends and relatives maybe also, create stress in the environment so it’s not beneficial for the person who would really need to have calm surroundings.*
P45	To have those I am close to around me	(8). *This might be important, but it may… Yes, but I mean, you just don’t know* [how it will be]. *If circumstances are favorable, that can be the case, but you can die alone also, if you die quickly, and so that’s that.*	(8). *Yes, I would really like some of them to be there so they can see what it’s like when someone dies, they’ve never experienced that, I think.*
*Reasoning about changed card choices*			
P42	Not being a burden to my family	(8). *No, you don’t want that. You just have to hope there’ll be room in a care facility when it’s needed.*	(−). *Yes, that’s important, that you’re not* [a burden]. *For example, when you start to be in such bad shape that you need to live in a care facility, that you really get a place there.*
P18	To be mentally aware	(−). *To be mentally aware, that also feels important. I’ve thought about it when I’ve worked with those who are both very physically ill but mentally aware, and then I’ve worked with those who have a serious dementia but a healthy body, and that’s worse, as I see it. Because if you are really sick you can still communicate how you want things to be.*	(1). *To be mentally aware, yes please* [short pause]. *Yes, if I can wish. Because it is…that is, my experience is like this, if your mind is clear and your body is sick, you can still take things in and you can understand. You have a really hard time. But to not be mentally aware, to be…* […] *I think that seems like it’s worse.*
P10	To die at home	(−). *I absolutely don’t want to be at one of those…yes, like an old age home or…* […] *And then it says it’s rehabilitation. I’m so* (curses) *tired of that, and I don’t want* [laughs]. […] *And I want to live at home, yes, that’s what I want as long as possible* […] *And I want to participate in the decision about when and where I will move, yes, that’s what I want. And if I’m in the kind of shape where I can’t, then I just want to die.*	(2). *I would prefer not to be cared for at all. And to die at home. I really don’t have any desire to die in some hospital you know.*
P53	To keep my sense of humor	(−). [No reasoning recorded].	(7). *This came about one evening when I came home, and was both angry and sad, and so… I called a neighbor who lives next door and we talked about it, and an image came into my mind that I told him about, and I laughed about it myself. And then afterwards I realized how good it was to remember your... to have your sense of humor left, because I remembered how it eased things and helped me.*
P33	To trust my doctor	(7). [No reasoning recorded].	(−). *Yes, you see what a difference…this about trusting the doctor. I think I had a good doctor at that time.*
P18	To be free of pain	(−). *Ehh…if I summarize this about having difficulty breathing, anxiety and worry, and being painfree, if I summarize it by saying if I have confidence in my doctor, that he’ll fix that, then I remove these three cards.*	(3). *As I said, if I keep these five physical cards* [To be free of pain, Not being short of breath, To be clean and neat, To be free of anxiety, To have human touch], *because I believe so. If this is taken care of and you know there is a plan. If I have more pain, then I’ll get more pain medicine… If I have more anxiety, then I can have some medicine or just someone to take care of me.*
P42	To have my funeral arrangements made	(10). *Yes, before I’ve gotten to the point about how I want to be buried, I was going to say, I hope you get care so that you’re not just lying there in pain.*	(−). *All that…that’s already planned you know.*
P53	To have an advocate who knows my values and priorities	(8). [No reasoning recorded].	(−). I: *That this is… it’s settled already.* M: *There’s someone that can speak for you?* I: *Oh yes, and we’ve spoken about this* [puts the card in the less important pile].

#### Reasoning about card choices that recurred over time in rankings

Similar reasoning about a recurring card statement in rankings at both T1 and T2 was generally characterized by the participant talking about a strong experience, opinion or habit about the preference in question (see illustrative quotes in Table 5). P9 had a profound childhood experience which the participant referred to in both interviews as influential on the choice of card statement ‘Not being short of breath’, while P18 chose the card statement ‘To have a human touch’ in both interviews based on a strong interest in tactile massage. For P53, the card statement ‘To pray’ was explained as self-evident in both interviews as it had been part of a life-long daily routine.

In rare cases, participants reasoned slightly differently over time about recurring card choices in rankings. For example, P7 ranked the card statement ‘To be cared for by staff I feel comfortable with’ in the top-10 in both interviews, but at T2 added nuances by problematizing the role of health care staff. P45 also showed modified reasoning about the card statement ‘To have those I am close to around me’ which seemed to concern the participant’s own comfort at T1, while at T2 those close to the participant were considered.

#### Reasoning about card choices that changed over time

There were numerous occasions when participants reasoned similarly about a DöBra card statement in both interviews, although ranked it among the top-10 preferences on only one occasion. As exemplified in Table 5, neither P42 nor P18 appeared to reason differently at T1 and T2 about card statements that were in the top-10 at only one timepoint. Similarly, P10 spoke extensively about a wish to remain at home at T1, although the statement ‘To die at home’ was a top-10 priority only at T2, following much briefer discussion.

There were also other variations in reasoning about card choices that changed in rankings over time. In some cases, the difference appeared to be in participants’ considerations of the relative importance of an item. For example, while P53 did not motivate why humor was not included in the top-10 ranking at T1, at T2 the participant described how a recent event had highlighted its’ importance. Change in importance of card items could also be due to external factors, as exemplified by P33 when retroactively explaining the top-10 ranking of ‘To trust my doctor’ at T1, as probably due to having a good doctor at the time.

In other situations, it appeared that the participant’s definition of a card item had changed over time. Participants sometimes considered interrelationships and overlap between card statements which led them to define and prioritize items differently in the two interviews. This is exemplified by P18 who ranked ‘To trust my doctor’ in the top-10 at T1 with the motivation that that choice would cover also pain, breathing and anxiety, card items which were included in the participant’s top-10 ranking at T2 only.

As exemplified by P42 and P53, another type of reasoning may indicate that if an issue had been resolved between interviews, it was considered less important at T2.

## Discussion

This study explores stability in ranking and reasoning about end-of-life values and preferences among community-dwelling older adults, using the Swedish DöBra cards to stimulate reflection and discussion in interviews 5.5–12 months apart. As might be expected, on a group level the most frequently prioritized card items at T1 were also those more likely to recur. Individual characteristics, i.e. demographic variables, change in self-reported health status, time elapsed or degree of card use between interviews, were not related to stability in card rankings. Through qualitative analysis, we found that consistent reasoning was not always paired with consistency in ranked card choices and changes in ranked card choices were not always related to changes in reasoning. Strong experiences or habits seemed influential when both reasoning and ranked card choices recurred over time, while changes in card choices could be explained by participants’ changing views of the definition or relative importance of a card item between timepoints.

In this study, a median stability of individual, ranked future end-of-life care preferences and priorities of 60% was found. This is comparable to that found in other studies measuring GoWish card ranking stability over time [22, 23]. Our findings differ somewhat from Delgado-Guay et al’s U.S. study [23], where the two most frequently ranked cards in the top-10 at both timepoints were related to religion, while in this Swedish study, conducted in a country known to be secular [34], on group level the top two ranked cards were related to physical comfort. In both studies, the card statement ‘to have those I am close to/my family with me’ was in third place in these rankings. However, in Delgado-Guay et al.’s study [23], it is not possible to determine whether the same individual ranked the card item in both interviews, which is a strength of the study presented here. As we have previously reported [25, 35], the relatively frequent use of wild card formulations about assisted dying in this sample is unique for studies using the GoWish cards. The present study contributes with knowledge about how preferences related to assisted dying also are subject to change over time.

The concept of response shift may be helpful in understanding these data. Response shift is defined as changes in respondents’ internal standards (“recalibration”), values (“reprioritization”), and/or definition of the target concept (“reconceptualization”) [36]. While we have shown examples of both reconceptualization and reprioritization, these data do not allow stringent investigation of recalibration. However, the lower rate of consistency among cards ranked lower in the top-10 at T1 compared to T2 (Fig. 2) may suggest a form of recalibration. It is possible that a card item may maintain both its definition and level of importance but no longer be in the top-10 if other card items have become more important, thus expanding the scale.

Preston et al. [37] suggest that in considering response shift, end-of-life clinicians should devote attention to exploring individuals’ ‘anchor values’. We found that profound values/preferences connected to strong habits or important life experiences were more likely to be stable over time, and when these clearly resonated with a card statement, that card would typically be prioritized in the top-10 ranking at both timepoints. As suggested also by our previous work [35] and other studies [38, 39], these findings provide further support for an iterative process of ACP, focusing on conversations about individuals’ anchor values rather than solely documenting medical treatment preferences. Others in the field [40, 41] argue for the importance of timely and repeated ACP discussions to allow for both advance as well as in the moment decisions.

## Strengths and limitations

Strengths of this study thus include the combination of exploring DöBra card rankings as well as underlying reasoning about end-of-life preferences over time, which furthers knowledge on the dynamics between values and preferences in end-of-life decision-making [42, 43]. Other strengths are the longitudinal design with community-dwellers in a natural setting with end-of-life preferences defined broadly, design choices also suggested by others [11, 38], as opposed to researcher-formulated hypothetical illness scenarios. Limitations include a larger risk of a type II error due to the relatively small sample size. The heterogeneity of the sample limits drawing conclusions based on statistical findings; however, our findings suggest that individual variation in card rankings would in itself discourage drawing such conclusions.

As self-reported health status may also be subject to response shift, exploring stability in relation to illness or life events would have been desirable. However, the lack of comprehensive, systematic data about possible illness progression or other life events between interviews in these data limits our ability to relate changes in preferences to such events. We did note that while two of the six participants lost to attrition reported their health state to be ‘neither good nor poor’ at the 1st interview, as group sizes were small we refrain from drawing conclusions based on this. Further research on end-of-life values and preferences in community-dwellers beyond the time-period reported on in this study would be valuable to increase knowledge about if, how, and when end-of-life values and preferences change over time.

In conclusion, our study suggests that there are different aspects that are indicative of stability of older adults’ values and preferences for future end-of-life care. Based on an intuitive ACP conversation-based card game, selection and ranking of the most important card items is relatively stable over time albeit with large variation between individuals. The values and underlying reasoning that participants used to motivate their choices appear more stable than ranking of card choices. We thus conclude that concurrent conversation-based exploration is a more comprehensive indicator of an individual’s end-of-life values and preferences over time than ranking of cards alone.

## Supplementary Information

**Additional file 1.** Overview of participants’ card choices.

**Additional file 2.** Supplemental demographic information.

## Data Availability

The datasets analyzed during the current study are not publicly available due to restraints of the ethical permit, some data may be available from the corresponding author upon reasonable request.

## Abbreviations

ACP: Advance care planning
SweACP: “Advance care planning in Sweden” (project name)

## Footnotes

1 DöBra is a Swedish pun, meaning both ‘dying well’ but also ‘awesome’.
