# Supplementary material for: Same, same, but different? A longitudinal, mixed-methods study of stability in values and preferences for future end-of-life care among community-dwelling, older adults
Source: BMC Palliat Care. 2021 Sep 22;20:148. doi: 10.1186/s12904-021-00839-7 (PMC8459471; doi:10.1186/s12904-021-00839-7)
Supplement: Supplementary file 2 — Additional file 2. Supplemental demographic information. [file 12904_2021_839_MOESM2_ESM.docx]

*Characteristics of original participants not included in analysis (N = 13)*

| **Characteristics** | **Attrition (N = 6)**  **N (%)** | **Incomplete records (N = 3)**  **N (%)** | **Excluded person from couple**  **(N = 4)**  **N (%)** |
| --- | --- | --- | --- |
| *Age*, median (range) | 75 (63-80) yrs | 73 (65-83) yrs | 68 (66-74) yrs |
| *Gender* |  |  |  |
| Female  Male | 5 (83,3)  1 (26,9) | 1 (33,3)  2 (66,7) | 2 (50,0)  2 (50,0) |
| *Living situation*  Spouse  Alone  With children | 3 (50,0)  3 (50,0)  0 (0,0) | 2 (66,7)  1 (33,3)  0 (0,0) | 3 (75,0)  1 (25,0)  0 (0,0) |
| *Education*  University  High school  Elementary school  Other | 2 (33,3)  2 (33,3)  2 (33,3)  0 (0,0) | 0 (0,0)  1 (33,3)  1 (33,3)  1 (33,3) | 3 (75,0)  1 (25,0)  0 (0,0)  0 (0,0) |
| *Employment status*  Retired  Employed, part-time  Retired, working part-time  Student, full-time  Employed, full-time | 6 (100)  0 (0,0)  0 (0,0)  0 (0,0)  0 (0,0) | 3 (100)  0 (0,0)  0 (0,0)  0 (0,0)  0 (0,0) | 3 (75,0)  1 (25,0)  0 (0,0)  0 (0,0)  0 (0,0) |
| *Self-assessed health status*  Good  Neither good nor poor  Poor | 4 (66,7)  2 (33,3)  0 (0,0) | 3 (100)  0 (0,0)  0 (0,0) | 3 (75,0)  1 (25,0)  0 (0,0) |
